# Reported drug spectrum and disproportionality signals for malignant neoplasm progression in FAERS: a real-world pharmacovigilance study

**DOI:** 10.3389/fphar.2026.1814403

**Published:** 2026-04-21

**Authors:** Yinghao Liu, Miao Zeng, Mingying Zhang, Hongxiang Xu, Xiaoyu Li, Jun Zhang

**Affiliations:** 1 National Clinical Research Center for Children and Adolescents’ Health and Diseases, Chongqing, China; 2 Department of Surgical Oncology Children’s Hospital of Chongqing Medical University, Chongqing, China; 3 Ministry of Education Key Laboratory of Child Development and Disorders, Chongqing, China; 4 CountryChongqing Key Laboratory of Child Rare Diseases in Infection and Immunity, Chongqing, China

**Keywords:** disproportionality analysis, drugs, FAERS, JADER, malignant neoplasm progression, pharmacovigilance, spontaneous reporting systems

## Abstract

**Objective:**

Using malignant neoplasm/tumour progression as the endpoint, we screened FAERS for disproportionate reporting signals, characterized the drug spectrum represented in progression-related reports, and prioritized drug–event pairs for further evaluation rather than causal inference.

**Methods:**

Publicly available reports from FAERS (2004Q1–2024Q4) and JADER (2004–2024) were analyzed. After FDA-recommended deduplication, malignant tumor progression was identified using the MedDRA Preferred Terms “Malignant neoplasm progression” and “Tumour progression” (version 26.1). Primary and secondary suspect drugs were standardized to generic names using MedEx. Signals were detected by disproportionality analysis using the reporting odds ratio (ROR), with positive signals defined as a report count ≥3 and a lower 95% confidence interval bound >1. The same analytical pipeline was applied to JADER for external validation.

**Results:**

FAERS contained 321,020 progression-related reports; 84,977 unique cases remained after deduplication, rising over time (notably after 2018) with severe outcomes (death 27.63%, hospitalization 13.66%). Among the top 50 drugs, 92% were antineoplastic/immunomodulating agents; nivolumab, pembrolizumab, enzalutamide, everolimus, and osimertinib were most frequently reported. 49 drugs showed positive ROR signals (highest: afatinib, gefitinib, osimertinib); 35/49 were replicated in JADER (8,929 cases).

**Conclusion:**

Progression-related reporting signals were concentrated mainly in immunotherapies and targeted agents. Although disproportionality analysis does not establish causality, these findings may help prioritize drug–event pairs for further investigation and highlight the need for more standardized reporting, as well as confirmatory epidemiologic and mechanistic studies.

## 1 Introduction

Malignant tumor progression often determines the course of treatment and patient outcomes. Globally, cancer is a leading cause of death. In 2020, nearly 10 million deaths were attributed to cancer, accounting for about one in six deaths worldwide (Sung et al., 2021). Even in high-income countries such as the United States, cancer remains the second leading cause of death after heart disease (Siegel et al., 2025). Treatment options have expanded in recent years. Immune checkpoint inhibitors (ICIs) are widely used in solid tumors and can improve survival in specific patients (Sharma et al., 2023). More than ten PD-1/PD-L1 or CTLA-4 antibodies have been approved worldwide for cancers such as lung cancer and melanoma (Tay-Teo et al., 2025). Yet one question remains: does improved efficacy mean that the risk boundary is equally clear? Atypical outcomes have been observed. Some patients develop unexpectedly rapid tumor worsening after treatment, termed hyperprogressive disease (HPD) (Adashek et al., 2020a). HPD is often described as a sudden acceleration of tumor growth kinetics during immunotherapy, accompanied by rapid clinical decline. It is uncommon but clinically serious (Leake, 2023). Systematic reviews report HPD rates of about 9.9%–18.06% across studies (Zhao et al., 2022). Patients with HPD have poorer outcomes than those with conventional progression, including shorter median overall survival (Zhao et al., 2022). The mechanism behind this type of atypical acceleration remains unclear, but there are still several factors have been linked to, including older age, liver metastases, elevated LDH, and low PD-L1 expression (Nishimura et al., 2024; He et al., 2023). Baseline immune features and genomic alterations, such as specific T-cell phenotypes and MDM2/MDM4 amplification, have also been proposed (Arasanz et al., 2020; Sun et al., 2024). For these reasons, abnormal progression has attracted increasing attention from both oncology and regulatory communities.

“Malignant neoplasm progression” is rarely listed as a typical adverse reaction in product labels. It is more often interpreted as lack of benefit or part of the natural course of cancer, rather than a direct toxic effect of a drug. Yet within the scope of this study, one question is hard to ignore: if progression is reported disproportionately often for a specific drug, should it not be treated as a potential safety clue (Noguchi et al., 2021)? Against this debated boundary of endpoint interpretation, large spontaneous reporting systems provide a useful, hypothesis-generating lens.

FAERS is among the largest spontaneous adverse event reporting systems worldwide, with more than ten million accumulated reports (Khaleel et al., 2022). Submissions come from healthcare professionals, patients, and manufacturers, supporting post-marketing safety monitoring in real-world settings (Ha et al., 2021). Compared with clinical trials, FAERS spans broader populations and longer time periods, which increases the chance of capturing rare, serious, or previously under-recognized events (Potter et al., 2025). Prior FAERS-based analyses have reported potential signals for several drug classes, including proton pump inhibitors and targeted therapies (Wang et al., 2025a; Li et al., 2025).

However, malignant neoplasm progression has seldom been treated as a primary endpoint in systematic mining. In many oncology safety analyses, progression or lack of benefit is excluded as a confounding outcome (Tan et al., 2025). However, the excluded information may also contain clues indicating unusually rapid deterioration. To address this gap, we screened drugs reported with malignant neoplasm progression in FAERS, quantified disproportionality signals, and then examined the same signals in JADER to assess robustness. The goal was not to claim causality, but to identify drug–event pairs with concentrated progression reporting and to provide risk hypotheses for regulators and clinicians.

## 2 Materials and methods

### 2.1 Data source and extraction

We used publicly available reports from the FDA Adverse Event Reporting System (FAERS, 2004Q1–2024Q4) and the Japanese Adverse Drug Event Report database (JADER, 2004–2024). FAERS quarterly files were downloaded from the official FDA website, imported into a relational database, and linked across the DEMO, DRUG, and REAC tables using PRIMARYID. Case-level deduplication was performed according to FDA recommendations: when multiple records shared the same CASEID, the most recent version was retained based on FDA_DT, and when FDA_DT was identical, the higher PRIMARYID was retained.

### 2.2 Endpoint definition

The endpoint was defined using MedDRA Preferred Terms (PTs): “Malignant neoplasm progression” and “Tumour progression” (MedDRA version:26.1). Cases were identified exclusively from the REAC table. Reports containing either PT were defined as cases, and all other deduplicated reports were treated as non-cases. Outcome definition was based only on REAC-coded events rather than indication or medical history fields.

### 2.3 Drug definition

Drug exposure was identified from the DRUG table. We used only primary suspect (PS) as the main analysis. When multiple suspected drugs were reported in one case, each drug was included separately in the corresponding drug-specific analysis. Drug names were standardized to generic names using MedEx before analysis.

### 2.4 Disproportionality analysis

Disproportionality analysis is commonly used to screen for disproportionate reporting patterns in large pharmacovigilance databases (Barcelos et al., 2019; Beau-Lejdstrom et al., 2019). We considered Proportional Reporting Ratio (PRR), Reporting Odds Ratio (ROR), Information Component (IC), and Empirical Bayes Geometric Mean (EBGM), and selected ROR as the primary metric based on its prior performance and interpretability in this setting (Hoffman et al., 2012; Lee et al., 2020; Ji et al., 2022). For each drug, a 2 × 2 contingency table was constructed from the deduplicated database, comprising reports with the target drug and the endpoint, reports with the target drug without the endpoint, reports without the target drug but with the endpoint, and reports with neither. Thus, the comparator for each drug consisted of all other reports not involving that drug as a suspected drug. Drug–event pairs with fewer than 3 reports were excluded. A positive signal was defined as a report count ≥3 and a lower 95% confidence limit of the ROR >1. Higher ROR values indicate stronger disproportionate reporting rather than stronger evidence of causality. For external cross-validation, the same endpoint definition, drug name normalization, and signal criteria were applied in JADER.

### 2.5 External validation

JADER was processed using the same analytical pipeline, including endpoint definition, suspected-drug selection, drug name normalization, and ROR-based signal criteria.

Our preliminary research results, which have been shared as a preprint before (Liu et al., 2025), present a preliminary correlation analysis of adverse events related to malignant tumor progression in the FAERS database.

## 3 Results

All findings reported below should be interpreted as hypothesis-generating pharmacovigilance signal-detection results from spontaneous reporting data. They describe reporting patterns and disproportionality signals rather than evidence of causal effects or comparative clinical risk.

### 3.1 Trend and gender-age analysis analysis

**FIGURE 1:**
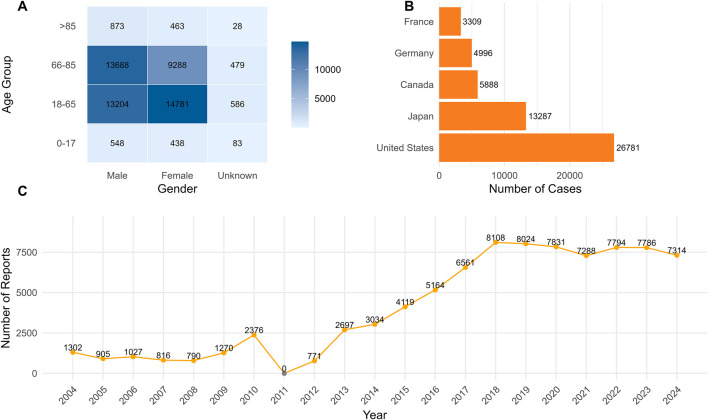
Clinical characteristics of reported cases of malignant tumor progression. **(A)** Heat map of Malignant Neoplastic Progression cases classified by age and gender; **(B)** Geographical distribution of Malignant Neoplastic Progression cases; **(C)** Annual Trends of Malignant Neoplastic Progression Report (2004–2024).

From Q1 2004 to Q4 2024, The FAERS database reports 321,020 cases related to malignant neoplasm progression were identified, anhlad 84,977 unique cases remained after deduplication. Report counts increased over time, with a steeper rise after 2018 (Figure 1C). In spontaneous reporting systems, shifts in drug exposure, reporting intensity, and coding practices can occur together, so time trends are best treated as context rather than direct evidence of increased risk. One unexpected finding was that no entries for this endpoint were captured for 2011 in our extracted dataset.

After excluding records with missing age or gender information, a total of 54,459 cases were available for analysis. Most patients were 18–65 years (52.46%, n = 28,571) or 66–85 years (43.07%, n = 23,455) (Figure 1A). Patients aged ≤17 years and ≥86 years accounted for 1.96% and 2.50%, respectively. Females accounted for 45.85% (n = 24,970) and males for 51.99% (n = 28,313); sex was missing in 2.16%. The largest number of reports came from the United States (31.52%), followed by Japan (15.64%), Canada (6.93%), Germany (5.88%), and France (3.89%) (Figure 1B). Notably, serious outcomes were common, including death (27.63%,n = 23,478) and hospitalization (13.66%,n = 10,755). However, in severe clinical situations, disease progression is more likely to be reported, which should be taken into account when interpreting study results.

### 3.2 Drug distribution and therapeutic spectrum

**FIGURE 2:**
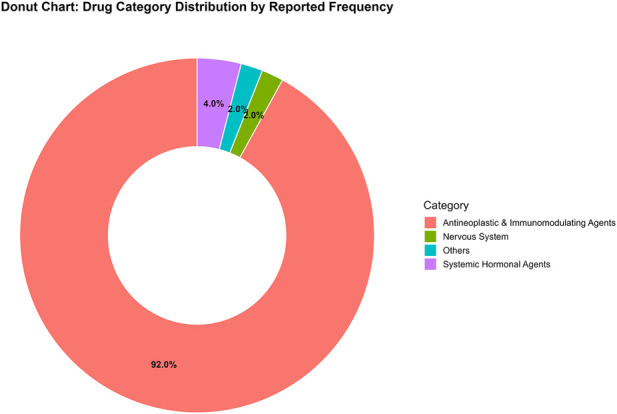
Classification of top 50 drugs associated with the malignant tumor progression.

Among the 50 prioritized drugs, antineoplastic and immunomodulating agents comprised 92% (46/50). Among the 46 drugs, targeted therapies accounted for the largest proportion (23/46, 50.0%), followed by cytotoxic chemotherapy (11/46, 23.9%), endocrine therapy (5/46, 10.9%), immunotherapy (4/46, 8.7%), and cellular/immunomodulatory therapies (3/46, 6.5%) (Figure 2). The top ten reported suspected drugs were nivolumab, pembrolizumab, enzalutamide, everolimus, osimertinib, imatinib, cabozantinib, octreotide, letrozole, and erlotinib. Importantly, high report volume does not equate to high risk; it can also reflect high exposure and broad use across indications.

### 3.3 Disproportionality analysis and external validation

**FIGURE 3:**
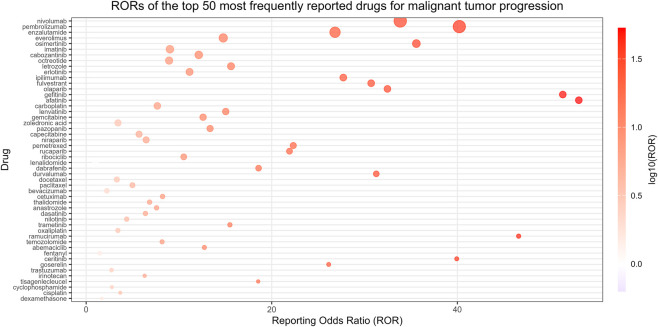
The distribution of ROR signal intensity of the top 50 drugs related to the progression of malignant tumors.

To evaluate the potential association between drugs and malignant tumor progression, a disproportionality analysis was conducted on the top 50 drugs ranked by reporting frequency. ROR ranged from 1.01 to 53.09. Positive signals were observed for 49 drugs (≥3 reports and lower 95% CI of ROR >1). The strongest signals were for afatinib (ROR 50.09), gefitinib (ROR 51.36), and ramucirumab (ROR 46.61) (Figure 3; Table 1).

**TABLE 1:** Drug information in FAERS and JADER.

Drugname	FEARS			JADER		
	Number of reports	1	ROR(95%Cl)	Number of reports	ROR(95%Cl)	ATCclassification
Afatinib	1561	53.09	53.09 (50.41-55.92)	120	20.07 (16.67–24.16)	Targeted therapies
Gefitinib	1570	51.36	51.36 (48.78-54.09)	64	4.29 (3.35–5.50)	Targeted therapies
Ramucirumab	542	46.61	46.61 (42.71-50.86)	93	3.83 (3.12–4.70)	Targeted therapies
Pembrolizumab	7216	40.21	40.21 (39.23-41.22)	3563	29.64 (28.56–30.75)	Immunotherapy
Ceritinib	494	39.94	39.94 (36.46-43.74)	60	28.27 (21.68–36.86)	Targeted therapies
Osimertinib	2291	35.58	35.58 (34.10-37.13)	149	7.67 (6.52–9.03)	Targeted therapies
Nivolumab	7426	33.85	33.85 (33.04-34.68)	414	2.30 (2.09–2.54)	Immunotherapy
Olaparib	1581	32.47	32.47(30.86-34.16)	333	26.03 (23.25–29.13)	Targeted therapies
Durvalumab	1068	31.27	31.27 (29.40-33.25)	262	8.08 (7.14–9.14)	Immunotherapy
Fulvestrant	1626	30.71	30.71 (29.21-32.29)	179	14.05 (12.09–16.34)	Endocrine therapy
Ipilimumab	1658	27.71	27.71 (26.37-29.12)	181	1.66 (1.44–1.92)	Immunotherapy
Enzalutamide	4784	26.82	26.82 (26.04-27.63)	39	2.62 (1.91–3.60)	Endocrine therapy
Goserelin	492	26.14	26.14 (23.88-28.61)	31	4.17 (2.92–5.94)	Endocrine therapy
Pemetrexed	1260	22.32	22.32 (21.09-23.62)	215	5.05 (4.41–5.78)	Cytotoxic chemotherapy
Rucaparib	1157	21.91	21.91 (20.66-23.24)	—	—	Targeted therapies
Dabrafenib	1077	18.58	18.58 (17.48-19.75)	88	15.63 (12.61–19.38)	Targeted therapies
Tisagenlecleucel	385	18.54	18.54(16.75-20.52)	—	—	Cellular/immunomodulatory therapies
Letrozole	1788	15.61	15.61 (14.89-16.37)	125	8.87 (7.42–10.60)	Endocrine therapy
Trametinib	583	15.49	15.49 (14.27-16.82)	88	14.82 (11.95–18.36)	Targeted therapies
Lenvatinib	1448	15.04	15.04 (14.27-15.85)	1017	21.55 (20.19–22.99)	Targeted therapies
Everolimus	2759	14.77	14.77 (14.22-15.35)	324	10.55 (9.44–11.80)	Targeted therapies
Pazopanib	1345	13.36	13.36 (12.65-14.10)	160	11.57 (9.87–13.55)	Targeted therapies
Abemaciclib	532	12.74	12.74 (11.69-13.89)	17	2.19 (1.36–3.53)	Targeted therapies
Gemcitabine	1433	12.6	12.60 (11.96-13.28)	231	4.51 (3.96–5.14)	Cytotoxic chemotherapy
Cabozantinib	2167	12.14	12.14 (11.63-12.67)	52	2.43 (1.85–3.20)	Targeted therapies
Erlotinib	1757	11.14	11.14 (10.63-11.69)	14	0.76 (0.45–1.28)	Targeted therapies
Ribociclib	1148	10.52	10.52 (9.92-11.15)	—	—	Targeted therapies
Imatinib	2173	9.03	9.03 (8.65-9.42)	189	5.86 (5.07–6.77)	Targeted therapies
Octreotide	1941	8.94	8.94 (8.55-9.36)	74	11.52 (9.13–14.54)	Systemic hormonal agents
Cetuximab	645	8.24	8.24 (7.62-8.90)	18	12.71 (7.93–20.38)	Targeted therapies
Temozolomide	533	8.19	8.19 (7.51-8.92)	23	1.40 (0.93–2.11)	Cytotoxic chemotherapy
Carboplatin	1555	7.67	7.67 (7.29-8.07)	649	4.82 (4.46–5.22)	Cytotoxic chemotherapy
Anastrozole	608	7.59	7.59 (7.00-8.22)	36	4.25 (3.06–5.91)	Endocrine therapy
Thalidomide	615	6.84	6.84 (6.31-7.40)	—	—	Cellular/immunomodulatory therapies
Niraparib	1278	6.47	6.47 (6.12-6.83)	2	0.16 (0.04–0.63)	Targeted therapies
Dasatinib	604	6.39	6.39 (5.90-6.93)	20	1.11 (0.72–1.73)	Targeted therapies
Irinotecan	385	6.31	6.31 (5.70-6.97)	41	0.59 (0.43–0.80)	Cytotoxic chemotherapy
Capecitabine	1319	5.7	5.70 (5.40-6.02)	46	0.99 (0.74–1.32)	Cytotoxic chemotherapy
Paclitaxel	795	4.99	4.99 (4.65-5.35)	340	3.49 (3.13–3.88)	Cytotoxic chemotherapy
Nilotinib	586	4.37	4.37 (4.03-4.74)	16	1.12 (0.68–1.83)	Targeted therapies
Cisplatin	353	3.67	3.67 (3.31-4.08)	388	3.64 (3.29–4.02)	Cytotoxic chemotherapy
Zoledronic acid	1348	3.43	3.43 (3.25-3.62)	115	3.11 (2.59–3.74)	Others
Oxaliplatin	549	3.42	3.42(3.14-3.72)	74	0.73 (0.58–0.92)	Cytotoxic chemotherapy
Docetaxel	858	3.31	3.31 (3.09-3.54)	147	4.96 (4.21–5.84)	Cytotoxic chemotherapy
Cyclophosphamide	356	2.78	2.78 (2.50-3.08)	109	0.98 (0.81–1.18)	Cytotoxic chemotherapy
Trastuzumab	431	2.74	2.74 (2.49-3.01)	2	4.05 (1.00–16.37)	Targeted therapies
Bevacizumab	679	2.23	2.23 (2.07-2.41)	109	0.90 (0.74–1.09)	Targeted therapies
Dexamethasone	331	1.69	1.69 (1.51-1.88)	702	4.73 (4.38–5.10)	Systemic hormonal agents
Fentanyl	495	1.47	1.47 (1.35-1.61)	29	1.46 (1.01–2.10)	Nervous system
Lenalidomide	1089	1.01	1.01 (0.96-1.08)	601	10.59 (9.75–11.50)	Cellular/immunomodulatory therapies

**FIGURE 4:**
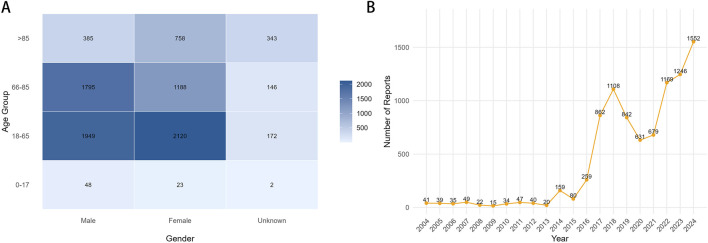
Clinical characteristics of reported cases of malignant tumor progression in Japan. **(A)** Heat map of Malignant Neoplastic Progression cases classified by age and gender; **(B)** Annual Trends of Malignant Neoplastic Progression Report (2004–2024).

**FIGURE 5:**
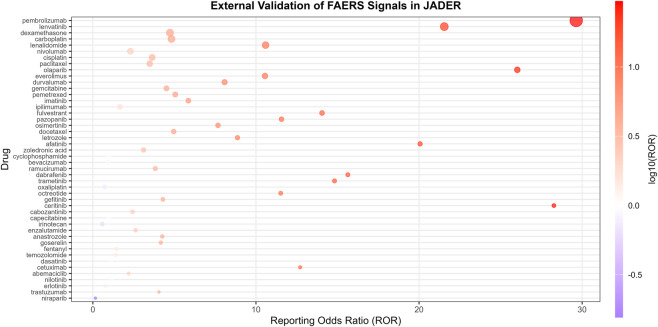
The FAERS database corresponds to the distribution of ROR signal intensity associated with malignant tumor progression in the JADER database for drugs.

In JADER, 8,929 related cases were identified, and demographic patterns were broadly similar (Figure 4). Of the 49 FAERS-positive drugs, 35 were replicated in JADER with consistent direction but different magnitude (Figure 5). Because the prescribing structure, launch timing, indication mix, and reporting behavior can all shape signal strength; under the constraints of spontaneous reports, these differences are best viewed as robustness cues rather than direct biological evidence.

## 4 Discussion

In the present study, we used FAERS and JADER to screen for disproportionate reporting signals related to malignant neoplasm progression, an unconventional endpoint that sits at the interface of safety reporting and disease course. Importantly, this study identifies progression-related reporting signals in spontaneous reporting systems rather than clinically adjudicated cases of hyperprogressive disease (HPD). Therefore, the HPD literature is cited here to provide clinical context for interpreting rapid-worsening narratives in oncology, but our findings should not be construed as evidence that the detected signals represent verified HPD events or as estimates of HPD incidence. Consistent signal patterns across FAERS and JADER also do not establish causality; rather, they suggest that certain drug–event pairs are repeatedly captured as “suspicious patterns” and thus warrant prioritization for further evaluation as hypothesis-generating findings.

From a therapeutic spectrum perspective, antineoplastic and immunomodulating agents comprised the largest share of the 50 drugs. Immune checkpoint inhibitors, particularly PD-1/PD-L1 antibodies, showed disproportionate reporting for tumor progression, which is directionally aligned with published clinical observations that a subset of patients may experience atypically rapid worsening during immunotherapy (Kim et al., 2021; Zheng et al., 2023; Li et al., 2023). Nivolumab and pembrolizumab were among the drug–event pairs that warrant priority attention. However, spontaneous reporting data cannot resolve why some patients obtain durable benefit while others worsen rapidly, nor can it delineate patient-level risk boundaries (Zhao et al., 2022; Park et al., 2021). Since HPD was described by Champiat et al. (2017), retrospective studies have reported rates of roughly 5%–15% among patients receiving immunotherapy (Ferrara et al., 2018; Kanjanapan et al., 2019). Ferrara et al. observed early accelerated tumor growth in about 16% of patients with advanced lung cancer treated with PD-1 inhibitors, with poorer early survival than chemotherapy controls (Ferrara et al., 2018). In hepatocellular carcinoma, Kim et al. reported an HPD rate of about 10% and suggested elevated LDH and liver metastases as potential risk factors (Nishimura et al., 2024; Li et al., 2023). These clinical and mechanistic observations provide plausibility for further investigation; nevertheless, our spontaneous-report endpoint is not equivalent to HPD definitions used in clinical studies, and key covariates needed to evaluate such risk factors are frequently missing or unstructured in FAERS/JADER. Accordingly, the immunotherapy-related signals observed here should be interpreted strictly as hypothesis-generating reporting patterns rather than evidence of clinically validated HPD in these datasets (Kim et al., 2019; Adashek et al., 2020b). Whether similar concerns extend to CTLA-4 inhibitors (e.g., ipilimumab) and emerging immunotherapy modalities remains an active area of research, and the boundary of evidence is not yet fully defined (Zang et al., 2020).

Disproportionate progression reporting was not restricted to immunotherapy. Signals were also observed for targeted agents and conventional chemotherapies. For example, in EGFR tyrosine kinase inhibitors, disproportionate reporting may plausibly arise in clinical scenarios where rapid deterioration follows acquired resistance or aggressive tumor biology, although spontaneous reports cannot disentangle resistance-driven disease course from treatment-related effects (Chaft et al., 2011; Wu and Shih, 2018). Signals observed for some cytotoxic agents (e.g., anthracyclines) should be interpreted with particular caution: higher signal metrics may reflect channeling to refractory populations with advanced disease, high tumor burden, or poor prognosis rather than a direct drug effect (Cavalli et al., 2025; Rafiyath et al., 2012). Overall, comparisons across drug classes are intrinsically confounded in spontaneous reporting systems, because therapies are used in different indications, disease stages, and lines of therapy, and the baseline probability of progression differs substantially across treated populations (Chen et al., 2024; Wu et al., 2025). Thus, class-level differences in reporting frequency or ROR are not interpretable as comparative clinical risk rankings.

Supportiv medications also appeared in the prioritized drug list, particularly dexamethasone. In oncology practice, dexamethasone is commonly used for supportive care, such as symptom control, antiemesis, edema management, and as part of combination regimens. Its presence in progression-related reports is therefore more likely to reflect clinical context—including advanced disease, palliative care, polytherapy, or reporting patterns—than a direct drug-related effect on tumor progression. This highlights the importance of distinguishing biologically plausible antitumor agents from supportive medications that may instead mark disease severity, treatment complexity, or reporting artifacts. For this reason, confounding by disease severity and treatment channeling should be considered part of the main interpretation of these findings, rather than only a limitation (Chen et al., 2024; Wu et al., 2025).

It is also necessary to clarify the endpoint itself. “Malignant neoplasm progression” is not a traditional adverse drug reaction and may reflect lack of benefit, resistance, delayed diagnosis of progression, or the natural history of advanced cancer rather than direct toxicity. As such, increased reporting does not imply causality, and the signals identified in this study should be interpreted as pharmacovigilance signals warranting further assessment, not as proof of drug-induced progression. A strength of pharmacovigilance mining is its scale and broad coverage, which can highlight concerns that may be underrepresented in clinical trials (Potter et al., 2025). Post-marketing surveillance has also proven valuable in identifying rare but serious immune-mediated toxicities for pembrolizumab and other agents, informing subsequent updates to labeling and monitoring considerations (Milutinovic et al., 2024). However, progression-related reporting patterns—especially those potentially reflecting lack of efficacy—require careful interpretation and should be confirmed in epidemiologic studies or curated real-world datasets before drawing causal inferences.

Taken together, our analysis provides a quantitative, reproducible signal-screening approach for an unconventional progression-related endpoint in spontaneous reporting systems. The appropriate implication of these findings is signal prioritization: drug–event pairs repeatedly flagged across databases may be prioritized for structured review and for targeted follow-up analyses, rather than being treated as early “risk detection” that directly mandates clinical management changes.

### 4.1 Limitations

Of course, our experiment has its limitations. First of all, spontaneous reporting systems such as FAERS are affected by under-reporting and selection bias. Only about 5%–10% of serious events may be reported (Hazell and Shakir, 2006). In oncology, tumor progression is often perceived as poor treatment efficacy or the natural course of disease rather than an adverse drug reaction, and therefore may be less likely to be reported, potentially leading to an underestimation of the overall reporting burden.

Meanwhile, we also conducted ranking analysis based on the ROR of all drugs and found that although some drugs had high ROR rankings and were roughly the same distribution as the most commonly reported drugs, some of these signals were based on relatively few reports and had large fluctuations in the 95% CI of ROR (Supplementary Figure 1). Due to the potential instability of disproportionate estimates derived from low reported counts and their susceptibility to random fluctuations, the main analysis focuses on the most commonly reported drugs.

More importantly, to investigate the absence of endpoint-related reports in 2011, we re-checked the original FDA quarterly source files and confirmed that data for the target endpoint were missing from the official 2011 files used for extraction. Therefore, the 2011 gap in our time series did not result from downstream deduplication or PT-based filtering, but reflected missing source data for that year. Temporal interpretation around this period should therefore be made with caution. Reporting bias also remains an important limitation. Notoriety bias may increase submissions for specific drug–event pairs following regulatory actions, major publications, or media attention, thereby affecting the stability and interpretation of disproportionality signals (Cutroneo et al., 2024; Gravel et al., 2024).

Furthermore, confounding—particularly confounding by disease severity—cannot be fully addressed in spontaneous reporting data. Treated populations may differ systematically in tumor aggressiveness, stage, metastatic burden, prior therapy, and prognosis (Chen et al., 2024). This issue is especially relevant when interpreting differences across drug classes (e.g., immunotherapies vs. chemotherapies), because these therapies are often used in distinct clinical settings and lines of therapy. For example, immunotherapies are frequently channeled to patients with advanced or refractory disease and limited treatment options, who may have a higher baseline risk of progression (Wu et al., 2025). Consequently, higher reporting frequencies or ROR observed for one drug class compared with another may partly reflect differences in underlying disease severity and treatment pathways (channeling/indication-related biases), rather than a drug-specific association. Because key clinical details (e.g., tumor type, stage, and line of therapy) are frequently missing, residual confounding is likely. Methods such as active-comparator–restricted disproportionality analysis may improve comparability within the same indication and reduce prescribing bias (Alkabbani and Gamble, 2023; Fusaroli et al., 2024); however, such restrictions cannot replace well-curated cohort data that capture staging and treatment trajectories.

Last but not least, disproportionality signals do not establish causality. Although cross-validation in JADER supports robustness, differences in prescribing patterns, launch timing, and reporting practices across countries may remain. These findings should therefore be treated as risk hypotheses and tested in cohort or case–control studies, or in curated real-world datasets, to confirm clinical relevance and quantify potential impact (Wang et al., 2025b).

## 5 Conclusion

Within the scope of this study, we used malignant neoplasm progression as a pharmacovigilance endpoint to mine FAERS and JADER and identified multiple drugs with disproportionate reporting for this outcome. The signals were not randomly distributed; instead, they clustered mainly in anticancer immunotherapies and targeted agents. Notably, disproportionality analysis does not establish causality, but it can help prioritize drug–event pairs that warrant closer monitoring, particularly when consistent reporting patterns are observed across databases. Even if such patterns are not emphasized in product labels, they may still add value for early safety surveillance.

These results also highlight a broader methodological issue in oncology pharmacovigilance: “disease progression” and “lack of benefit” may be variably captured and interpreted within spontaneous reporting systems, creating a grey zone between safety-related reporting and efficacy-related clinical course. Our findings therefore motivate further work on how progression-related concepts are coded and analyzed, with the aim of improving consistency and interpretability of this endpoint in real-world safety monitoring. However, any refinement of reporting and classification frameworks should be guided by validation studies and clinical context, given the inherent ambiguity of progression-related outcomes.

Future work should test these hypotheses in more comparable populations and with designs that better address confounding by disease severity and treatment indication. Prospective studies and well-designed real-world analyses are needed to assess the robustness of the identified signals and to clarify whether specific biological or clinical mechanisms underlie the observed reporting patterns. Improved linkage between pharmacovigilance systems and structured clinical sources (e.g., cancer registries and treatment pathway data) may enable more precise characterization of atypical progression patterns and support evidence-based interpretation of progression-related safety signals.

## Data Availability

The original contributions presented in the study are included in the article/Supplementary Material, further inquiries can be directed to the corresponding author.
