# Supplementary figures and images for: Reported drug spectrum and disproportionality signals for malignant neoplasm progression in FAERS: a real-world pharmacovigilance study
Source: Front Pharmacol. 2026 Apr 21;17:1814403. doi: 10.3389/fphar.2026.1814403 (PMC13139327; doi:10.3389/fphar.2026.1814403)

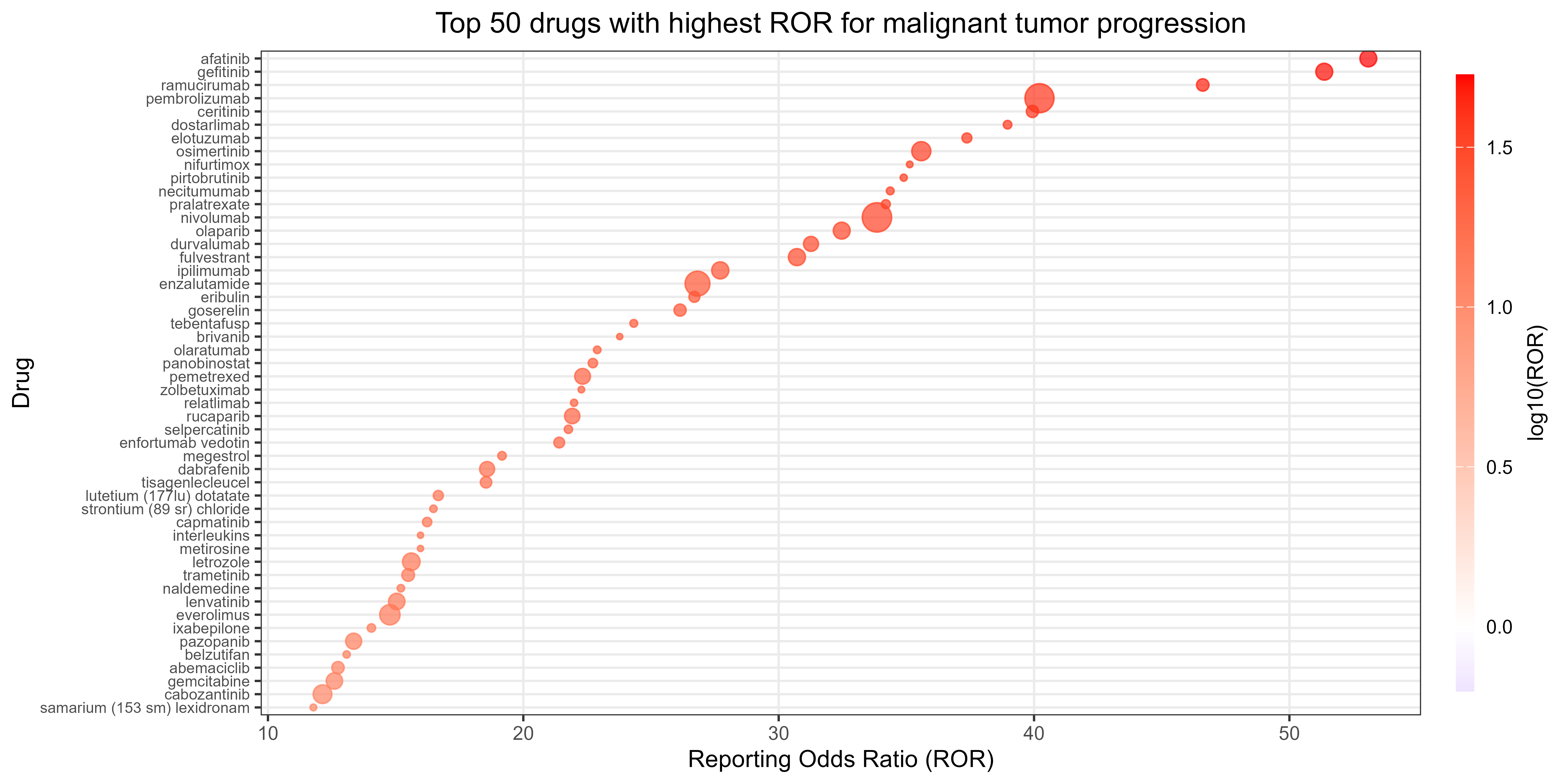

Supplement: Supplementary file 1 [file Image1.png]
